# Addition of vancomycin to cefazolin is often unnecessary for preoperative antibiotic prophylaxis during total joint arthroplasties

**DOI:** 10.1186/s42836-023-00222-2

**Published:** 2024-03-08

**Authors:** Sandeep S. Bains, Jeremy A. Dubin, Daniel Hameed, Zhongming Chen, Mallory C. Moore, Ashesh Shrestha, James Nace, Ronald E. Delanois

**Affiliations:** Lifebridge Health, Sinai Hospital of Baltimore, Rubin Institute for Advanced Orthopedics, 2401 West Belvedere Avenue, Baltimore, MD 21215 USA

**Keywords:** Vancomycin, Cefazolin, Antibiotics, Arthroplasty, MRSA

## Abstract

**Purpose:**

The gold standard to decrease total joint arthroplasty (TJA) periprosthetic joint infection (PJI) is preoperative antibiotic prophylaxis. Despite substantial prevention efforts, rates of PJIs are increasing. While cefazolin is the drug of choice for preoperative prophylaxis, adjunctive vancomycin therapy has been used in methicillin-resistant *Staphylococcus aureus* (MRSA) endemic areas. However, studies examining these combinations are lacking. Therefore, we sought to examine complications among vancomycin plus cefazolin and cefazolin-only recipients prior to primary TJA in a single institutional sample and specifically assessed: (1) microbiological aspects, including periprosthetic joint and surgical site infections, microbes cultured from the infection, and frequency of microbes cultured from nasal swab screening; (2) 30-day emergency department (ED) visits and re-admissions; as well as (3) associated risk factors for infection.

**Methods:**

A total of 2,907 patients (1,437 receiving both cefazolin and vancomycin and 1,470 given cefazolin only) who underwent primary TJA between 1 January 2014 and 31 May 2021 were identified. SSI and PJI as well as rates of cultured microbes rates were obtained through one year, those with prior nasal swab screening and 30-day re-admission were identified. Subsequently, multiple regression analyses were performed to investigate potential independent risk factors for PJIs.

**Results:**

There was no significant difference in the rates of SSI (*P* = 0.089) and PJI (*P* = 0.279) between the groups at one year after operation. Commonly identified organisms included *Staphylococcus* and *Streptococcus* species*.* The VC cohort did have a greater reduction of MRSA in the previously nasal swab-screened subset of patients. Multiple regression analyses demonstrated emergency as well as inpatient admissions as risk factors for PJI.

**Conclusions:**

Adjunctive vancomycin therapy offers increased protection against MRSA in previously screened individuals. However, those negative for MRSA screening do not require vancomycin and have similar protection to infection compared to recipients of cefazolin only in a high-powered single institution analysis in an MRSA endemic area.

## Introduction

Infections account for approximately 1.5% of all primary joint arthroplasty complications and lead to nearly 33% of all-cause revisions [1]. A significant number of these infected joints have led to disastrous outcomes, with an associated increase in financial burden [2]. Therefore, there continues to be a focus on the prevention and treatment of infections for total joint arthroplasty (TJA). The Centers for Medicare and Medicaid (CMS) initiated quality metrics for hospital-acquired infections with specific cost-related penalties. These regulations led to institutional medical surveillance and enforced preventative practices such as antimicrobial prophylaxis. Traditionally, surgeons preferred cefazolin for preoperative antibiotic prophylaxis [3]. However, due to the emergence of methicillin-resistant *Staphylococcus aureus* (MRSA), surgeons may choose vancomycin (VC) in addition to cefazolin.

Vancomycin alone does not provide broad anti-microbial coverage, thus still requiring another antibiotic, such as cefazolin. Although the coverage of these anti-microbials is vast, the paucity of literature supporting the combined effectiveness has led to a lack of consensus among surgeons [4, 5]. In the past decade, multiple studies compared dual antibiotic coverage with cefazolin alone [6–12]. A few studies demonstrated a nearly 10-fold decrease in surgical site infections (SSI) among vancomycin plus cefazolin prophylaxis recipients compared to cefazolin alone [6–9]. In contrast, other investigations found no difference in infection risk between vancomycin adjunct therapy versus cefazolin only [10–12]. Limitations of these studies include short follow-up time, small sample size, use of historical controls, and non-random patient selection that possibly result in selection bias. Given these limitations, further direct studies comparing vancomycin plus cefazolin versus cefazolin alone in a community-acquired MRSA (CA-MRSA) endemic area are warranted.

Despite the successful use of prophylactic antibiotics, there is a paucity of data on the incidence of infection with utilization of preoperative vancomycin plus cefazolin prior to TJA in MRSA endemic areas in a single institution study. Therefore, we sought to examine 90-day and one-year complications among vancomycin plus cefazolin versus cefazolin only recipients before primary TJA in a single-institutional sample and specifically assessed: microbiological aspects, including periprosthetic joint and surgical site infections, microbes cultured from the infection, frequency of microbes cultured from nasal swab screening; (2) 30-day emergency department (ED) visits and re-admissions; as well as (3) associated risk factors for infection.

## Methods

### Patient selection

A total of 2,907 patients (1,437 receiving both cefazolin and vancomycin, 1,470 cefazolin only) who underwent primary TJA between 1 January 2014 and 31 May 2021 at a single institution were identified. Inclusion criteria consisted of primary knee or hip prosthesis recipients from a CA-MRSA endemic community (Baltimore, MD, USA) who received adequately dosed vancomycin within one hour of incision for the dual antibiotic cohort as well as appropriately dosed cefazolin infusion within one hour of incision for both cohorts [13]. The use of antibiotics was based on surgical preference rather than baseline patient characteristics. The exclusion criteria were patients who had prior hardware in the joint of interest, patients scheduled for the same-day bilateral joint arthroplasty, allergies to either cefazolin or vancomycin, recipients of clindamycin for any reason, and patients who had lengths of stay (LOS) from hospital admission to discharge of greater than or equal to four days. The LOS restriction was implemented to exclude patients who were potentially in the surgical intensive care units, while patients who had longer LOS only due to requiring insurance authorization or awaiting rehabilitation placement were still included. Additionally, all patients received postoperative antibiotics as per the Surgical Care Improvement Project (SCIP) guidelines [14]. One senior joint fellowship-trained surgeon (R.E.D) performed the arthroplasty for those receiving VC prophylaxis, while the cefazolin only operations were conducted by two other senior joint fellowship-trained surgeons (J.N. and M.A.M.). Exemption status was provided by the institutional review board given the retrospective nature of the study.

### Patient demographics and baseline characteristics

Both groups had a mean age of 62 years. The overall mean body mass index (BMI) was 32.8 kg/m^2^ (range: 17.2 to 64.9) for VC and 32.9 kg/m^2^ (range: 16.2 to 71.5) for the cefazolin cohort. Both groups had similar male and female populations (*P* = 0.279). There was a statistically significant difference in race between cohorts (*P* < 0.05). African Americans comprised 48.4% of the VC cohort, whereas a larger population of Caucasian whites (50.7%) were in the cefazolin-only cohort (*P* < 0.006). The number of hips versus knees arthroplasties in either cohort was not significantly different, and, in fact, each cohort had approximately 50% knees as well as 50% hips (*P* = 0.914). Diabetes mellitus (24.1%), hypertension (47.0%), and chronic kidney disease (5.4%) were more common among the VC cohort (*P* < 0.014). Similar rates of tobacco users, substance abuse, chronic obstructive pulmonary disease, cardiac heart failure, and American Society of Anesthesiology (ASA) classes were seen in both cohorts (*P* > 0.097) (See Table 1).

**Table 1:** Demographics and baseline characteristics

Variable	**Vancomycin & Cefazolin (*n* = 1,437)**		**Cefazolin (*n* = 1,470)**		***P*-value**
	*n*	%	*n*	%	
Age (years)	62		62		
BMI (kg/m^2^)					
BMI < 20	16	1.1%	32	2.2%	0.020
20 < BMI < 30	543	37.8%	461	31.4%	< 0.001
30 < BMI < 40	546	40.0%	570	38.8%	0.508
BMI > 40	170	11.8%	180	12.2%	0.740
Gender					
Female	809	56%	801	54%	0.279
Male	628	44%	669	46%	0.279
Race					
American Indian or Alaska Native	4	0.3%	7	0.5%	0.394
Asian	6	0.4%	27	1.8%	< 0.001
Black or African American	695	48.4%	637	43.3%	0.006
White	554	38.6%	745	50.7%	< 0.001
Pacific Islander	1	0.1%	1	0.1%	0.999
Unknown	8	0.6%	22	1.5%	0.018
Multiple	31	2.2%	31	2.1%	0.853
Joint					
Hip	719	50.0%	732	49.8%	0.914
Knee	718	50.0%	738	50.2%	0.914
Alcohol Abuse	451	31.4%	860	58.5%	< 0.001
Tobacco Users	665	46.3%	714	48.6%	0.215
Substance Abuse	91	6.3%	94	6.4%	0.912
Diabetes mellitus	347	24.1%	298	20.3%	0.014
COPD	30	2.1%	34	2.3%	0.713
CHF	48	3.3%	59	4.0%	0.315
HTN	676	47.0%	603	41.0%	0.001
CKD	77	5.4%	51	3.5%	0.013
ASA Class					
1	36	2.5%	37	2.5%	0.999
2	814	56.6%	875	59.5%	0.113
3	577	40.2%	547	37.2%	0.097
4	10	0.7%	8	0.5%	0.485

*BMI* body mass index, *COPD* chronic obstructive pulmonary disease, *HTN* hypertension, *CKD* chronic kidney disease, *ASA* American Society of Anesthesiology, *CHF* congestive heart failure

The orthopaedic surgery department at our institution also implemented a community-acquired methicillin-resistant *Staphylococcus aureus* (CA-MRSA)-specific nasal swab screening. As a component of the chart review, the authors found an incidence rate of 7.5% (129 out of 1,719 screened patients) in those who were positive for MRSA tested by nasal swabbing. Furthermore, among the VC recipients, 1,112 patients were screened and 95 (8.5%) were positive for CA-MRSA, whereas, in the cefazolin cohort, 607 were screened and 9 (1.5%) were positive.

### Outcomes of interest

The primary outcome of this study was PJI and SSI. PJI was defined as an infection, based on Musculoskeletal Infection Society (MSIS) criteria, followed by a revision [15]. SSI was defined as apparent signs of superficial infection at the surgical site, on clinical examination (e.g., erythema, infected hematoma, abscess, superficial drainage), followed by antibiotic treatment and/or irrigation and debridement (I&D) when necessary. Several secondary outcomes examined included the specific microbe cultured, emergency department (ED) visits, re-admissions, and independent risk factors for infection. PJI and complication rates were obtained over a time of one year, along with 30-day ED and re-admission rates. SSI covers surgical site infection of all severities, including wound infection, infected postoperative seroma, infection, and inflammatory reaction resulting from the internal joint prosthesis, and incision and drainage of skin and subcutaneous tissue. PJI was defined by a deep joint infection requiring a surgical intervention to exclude any superficial wound complications, *as per* the MSIS criteria [15]. All PJI were confirmed by an infectious disease specialist and the attending surgeon. Acute infections within 3 to 6 weeks from the index procedure may be treated with debridement, antibiotics, modular head/liner exchange, and implant retention. Chronic infections may be treated with a traditional 2-stage exchange arthroplasty, in which an antibiotic spacer is placed, which is followed by a subsequent revision once the infection has been cleared.

### Data analyses

Patient data, including age, sex, comorbidities, and complications were compared using Student’s *t*-tests and Pearson’s chi-squared tests. Multiple regression analyses were performed to investigate potential independent risk factors for infection. ASA class ≤ 2, sex, alcohol abuse, substance abuse, tobacco use, congestive heart failure, chronic obstructive pulmonary disease, hypertension, diabetes mellitus, chronic kidney disease, ED visits, inpatient re-admission, type of surgery (knee versus hip arthroplasty) and preoperative VC versus cefazolin were the potential risk factors studied. Statistical analyses were conducted using SPSS software (Version 24, IBM, Chicago, IL, USA). Statistical significance was set to a *P*-value threshold of < 0.05.

## Results

### PJI, SSI, and microbe rates

The rate of PJI at 90 days (0.5% versus 0.3%) and one year (1.2% versus 0.8%) were higher in the cefazolin-only group than in the VC cohort, but the difference was not statistically significant (*P* > 0.279). Similarly, SSIs at 90 days (0.5% versus 0.2%) and one year (0.6% versus 0.2%) were greater in the cefazolin-only cohort, and the difference was getting close to significance (*P* > 0.089) (See Table 2).

**Table 2:** Outcomes and complications

**Variable**	Vancomycin & Cefazolin (*n* = 1,437)		Cefazolin (*n* = 1,470)		*P-*value
	*n*	%	*n*	%	
**30-day Complications**					
Emergency	60	4.2%	80	5.4%	0.131
Inpatient Readmission	53	3.7%	97	6.6%	< 0.001
**90-day Complications**					
PJI	5	0.3%	8	0.5%	0.394
SSI	3	0.2%	8	0.5%	0.172
**1-year Complications**					
PJI	12	0.8%	17	1.2%	0.279
SSI	3	0.2%	9	0.6%	0.089
**PJI Infecting Organism**					
MRSA	1	0.1%	2	0.1%	0.999
MSSA	1	0.1%	4	0.3%	0.229
*Serratia marcescens*	1	0.1%	0	0.0%	0.225
*Streptococcus agalactiae*	2	0.1%	0	0.0%	0.225
*Streptococcus viridans*	0	0.0%	1	0.1%	0.231
Polymicrobial	0	0.0%	2	0.1%	0.231
No growth	7	0.5%	8	0.5%	0.999
**SSI Infecting Organism**					
Corynebacterium	0	0.0%	1	0.1%	0.231
MSSA	0	0.0%	3	0.2%	0.089
Peptococcus species	0	0.0%	1	0.1%	0.231
Polymicrobial	2	0.1%	2	0.1%	0.999
No growth	1	0.1%	2	0.1%	0.999

*PJI* periprosthetic joint infection, *SSI* surgical site infection, *MRSA* methicillin resistant *Staphylococcus aureus*, *MSSA* methicillin sensitive *Staphylococcus aureus*

A sub-analysis was conducted and every sample of SSI or PJI was cultured in the operating room. Overall, among the PJIs at one year, the rate of methicillin-resistant *Staphylococcus aureus* (MRSA) infection was 8.33% (1 out of 12) for the VC cohort and 11.8% (2 out of 17) for the cefazolin cohort. Other predominate organisms isolated in the VC compared to cefazolin cohorts were methicillin-sensitive *Staphylococcus aureus* (MSSA) (8.33% versus 23.5%), *Streptococcus agalactiae* (16.7% versus 0%), and a mixture of multiple microbes (0% versus 11.8%). Additionally, 58.3% (7 out of 12) of the VC recipients and 47.1% (8 out of 17) of the cefazolin-only cohort had no bacterial growth, however, were positive for PJI based on the MSIS criteria. Furthermore, cultures from the SSI samples grew Corynebacterium (11.1%, 1 out of 9), MSSA (33.3%, 3 out of 9), *Peptococcus* species (11.1%, 1 out of 9), and a mixture of organisms (66.7% VC (2 out of 3) versus cefazolin (22.2%, 2 out of 9)) (Table 2).

Of those screened for CA-MRSA by way of nasal swabbing, three patients had PJIs. Two were positive for MRSA following TJA (one in the VC cohort and one in the cefazolin cohort). Since the VC cohort was 8.5% positive for CA-MRSA, while the cefazolin cohort was 1.5% positive, a reduction of 8.04% (8.1% positive via nasal swab screening minus 0.1% that had an MRSA PJI postoperatively) in the VC cohort and 1.3% (1.5% positive via nasal swab screening minus 0.2% that had a MRSA PJI postoperatively) was observed. For all the cases of PJI, in which no infecting organism growth was found, treatment included debridement, antibiotics, modular head/liner exchange, and implant retention. For all the cases of SSI, in which no infecting organism growth was found, treatment included wound drainage, debridement, and antibiotics.

### Emergency department visits and re-admissions

An association between emergency department (ED) visits or readmissions at 30 days and the type of antibiotic prophylaxis regimen administered was not apparent (*P* > 0.172). However, cefazolin recipients did have a higher rate of ED visits (5.4% (80 out of 1,470) versus 4.2% (60 out of 1,437)) and a higher rate of inpatient re-admission (6.6% (97 out of 1,470) versus 3.7% (53 out of 1,437)).

### Associated risks for infection

A multivariate regression model was used to identify risks for infection among the two cohorts. Overall, a significant association was not observed between the antibiotic prophylaxis regimen or type of surgery (knee versus hip arthroplasty) and the risk of infection (*P* > 0.117). Of note, substance abuse was at borderline as a significant risk for infection (*P* = 0*.*054), and ED visits and inpatient re-admission had an up to 11-fold increase in risk for infection (*P* ≤ 0.005). Other baseline characteristics did not confer a risk for infection (*P* > 0.201) (See Table 3).

**Table 3:** Multivariate logistic regression for all cause infection

**1-year**	**OR**^a^	**95% CI**	***P*-value**
Sex	1.01	0.53–1.94	0.973
ASA Class	1.65	0.83–3.30	0.153
VC vs. CF	1.47	0.76–2.86	0.253
TKA vs. THA	1.71	0.87–3.36	0.117
CHF	1.58	0.46–5.40	0.470
CKD	0.67	0.14–3.26	0.616
COPD	1.92	0.38–9.60	0.427
Diabetes Mellitus	1.43	0.66–3.10	0.369
HTN	0.80	0.39–1.64	0.547
Alcohol Use	1.28	0.61–2.68	0.513
Substance Abuse	2.62	0.98–6.98	0.054
Tobacco Use	1.56	0.79–3.07	0.201
Emergency	3.58	1.46–8.77	0.005
Inpatient Readmission	11.01	5.47–22.18	< 0.001

*OR* odds ratio, *95% CI* 95% confidence intervals, *VC* vancomycin and cefazolin, *CF* cefazolin, *ASA* American Society of Anesthesiologists, *CHF* congestive heart failure, *CKD* chronic kidney disease; chronic obstructive pulmonary disease, *HTN* hypertension, *TKA* total knee arthroplasty, *THA* total hip arthroplasty

^a^Referent group: Matched control without corresponding comorbidity

## Discussion

The cornerstone of total joint arthroplasty (TJA) infection reduction is antimicrobial prophylaxis. First and second-generation cephalosporins, such as cefazolin have been the antibiotic of choice due to the coverage necessary for skin flora. However, the rise of community-acquired methicillin-resistant *Staphylococcus aureus* (CA-MRSA) has driven surgeons to include vancomycin in addition to cefazolin. Therefore, we sought to compare VC and cefazolin as preoperative antibiotics in a CA-MRSA endemic community. Our results demonstrated no significant difference in prosthetic joint infection or surgical site infection (SSI) between preoperative antibiotic prophylactic measures with VC and cefazolin (*P* > 0.089). Microbes identified as the cause of infections included methicillin-resistant *Staphylococcus aureus* (MRSA) (VC cohort, 1 out of 12, 8.33% versus cefazolin cohort, 2 out of 17, 11.8%) and methicillin-sensitive *Staphylococcus aureus* (MSSA). Other cultured microbes included a mix of organisms and some cultures showed no bacterial growth (See Table 2). Additionally, the authors analyzed nasal swabs for MRSA screening and found that 2 out of 3 cases (66.7%) of postoperative infections did yield a positive result. However, the greatest reduction in positive results of postoperative MRSA infection in nasal swab screening was in the VC cohort (8.0%). Lastly, emergency department visits and inpatient visits were found to predispose patients to risk of PJI.

Our study is not without limitations. Due to the low incidence rate of infection (< 1.5%) following joint arthroplasty, a larger patient population would strengthen this study. Furthermore, the patient population in all three cohorts was operated on by three different surgeons at the same institution, raising the possibility of alterations in pre-, intra-, and postoperative practices. Nevertheless, all three surgeons are senior fellowship-trained joint arthroplasty orthopaedic surgeons that follow similar institutional guidelines. Since this study did not involve such preoperative laboratory as blood routines, IL-6, CRP, ESR, etc., we could not reach any conclusion about whether the postoperative SSI and PJI were related to the abnormality of preoperative laboratory tests or not. Another weakness is that not all patients underwent nasal swabbing, but this protocol was not fully adopted by the beginning of data collection. Despite these limitations, our finding that the addition of vancomycin may not decrease the risk for PJI can serve as adjunctive knowledge to orthopaedic surgeons. Although the preoperative infection risk in the cohorts could not be determined, we found similar baseline demographics, demonstrating characteristically similar patients who were undergoing treatment with either antibiotic. Additionally, the results and analysis of the microorganisms cultured as well as screening nasal swabs may allow surgeons to modify antibiotic prophylaxis plans accordingly. The strengths of our study lie in the analysis of the incidence of infection with utilization of preoperative vancomycin plus cefazolin administration prior to TJA utilizing large patient numbers at a single institution in a MRSA endemic area.

The reduced incidence of infection following surgery is greatly attributed to antibiotic prophylaxis. Since multiple organisms often contribute to skin flora, antibiotics with wide coverage are needed to prevent infection. Thus, first and second generation cephalosporins traditionally are the antibiotic of choice for preoperative prophylaxis. Cephalosporins target the cell wall synthesis of bacteria and inhibit cross-linking of peptidoglycan and can work on both aerobic and anaerobic microbes [16]. Additionally, these bactericidal antibiotics are resistant to beta-lactamase produced by the *Staphylococcus* species, making it an ideal defense against predominate organisms that may be the cause of infection [16]. However, the overuse of such antimicrobials has led to the evolution of *Staphylococcus* species to a specific strain expressing the *mecA* gene that provides resistance to beta-lactam-based antibiotics [17]. As these new strains emerged from areas outside of the hospital [that is, they were community-acquired methicillin-resistant *Staphylococcus aureus* (CA-MRSA)], surgeons opted to utilize vancomycin in addition to cefazolin as a regimen for antibiotic prophylaxis. Vancomycin prevents the synthesis of N-acetylmuramic acid and N-acetylgucosamine by binding to D-alanyl D-alanine, thus weakening the bacterial cell wall [18, 19]. Since these structures are only present in gram-positive bacteria, vancomycin has a narrow spectrum of use and is less ideal for appropriate skin flora coverage [18, 19]. Additionally, vancomycin has an extensive side-effect profile that may be life-threatening [20]. The limited use and the side-effect profile of vancomycin combined with the alarming rate at microbes are developing antibacterial resistance suggests vancomycin should not be administered to every patient undergoing a procedure, but only to those that are predisposed to MRSA infection.

Nasal swabbing remains an effective screening tool for the detection of MRSA in an otherwise healthy patient [21, 22]. In a retrospective review of Veterans Affairs (VA) medical centers from 1 January 2007 to 1 January 2018, Mergenhagen et al., [21] reported a specificity of 81.2% and a negative predictive value of 96.5% for ruling out MRSA infection following a nasal swab screening. In the present study, those who were positive for an MRSA nasal screening were not treated with nasal mupirocin and were the same patients who had a postoperative MRSA infection, regardless of antibiotic prophylaxis. Thus, screening and appropriate prophylactic measures may be more important than previously emphasized. This notion was further supported by the reduced MRSA infection (-8.0%) when those positive for MRSA screening (8.1%) were compared to their postoperatively infected counterparts (0.1%) in the VC cohort. In contrast, the same comparison within the cefazolin-only cohort yielded only minimal reduction. Similarly, multiple other studies have demonstrated a reduction in MRSA infection following screening with nasal swabs and appropriate treatment [6, 10, 23–26]. Therefore, preoperative vancomycin in addition to cefazolin may be warranted in a high-risk population.

With regard to the addition of vancomycin to cefazolin in a non-high-risk population, risks might outweigh benefits. Overall, the number of cases of postoperative PJIs and SSIs at one year did not significantly differ between the VC and cefazolin cohorts (*P* > 0.089). Additionally, all organisms cultured were sensitive to cefazolin and in some cases, their microbes were only sensitive to cefazolin (gram-negative organisms) thus eliminating the need for the addition of vancomycin. Multiple other studies have demonstrated similar results [6, 9–11, 27–29]. Sewick et al. [9] retrospectively reviewed 1,828 primary total joints from 1 September 2008 to 31 December 2010 and demonstrated no association between antibiotic prophylaxis regimen (VC versus cefazolin) and postoperative infection. However, a reduction in MRSA was apparent with the preoperative addition of vancomycin. Limitations of these previous studies included small sample size, resultant failure to achieve clinical significance, and selection bias resulting from high-risk patients who were predetermined to be given vancomycin. Clinically, the administration of dual antibiotics compared to cefazolin alone does not seem to yield any difference in PJI and SSI rates. However, a subset of patients that may be at high risk for MRSA infections would potentially benefit from the single dose of vancomycin in addition to cefazolin preoperatively.

## Conclusion

Adjunctive vancomycin therapy appears to offer similar protection compared to cefazolin-alone prophylaxis prior to total joint arthroplasties at a single institution in an MRSA endemic area. Further research is warranted to elucidate the role of judicious adjunctive vancomycin therapy in MRSA-endemic areas. The authors recommend knowing your institute’s antibiogram and performing nasal swabs on all patients to test for MRSA preoperatively. Those who are colonized with MRSA should be treated with vancomycin in addition to cefazolin.

## Data Availability

Not applicable.
